# A system review of central nervous system tumors on children in China: epidemiology and clinical characteristics

**DOI:** 10.1186/s12885-024-11883-w

**Published:** 2024-01-27

**Authors:** Bing Yao, Hongying Wang, Xiaomei Wu, Chenyu Wang, Tao Tang, Wenxiu An, Bo Zhu

**Affiliations:** 1 Department of Neurosurgery, Cancer Hospital of China Medical University/Liaoning Cancer Hospital & Institute, Shenyang, Liaoning Province China; 2 Department of Cancer Prevention and Treatment, Cancer Hospital of China Medical University/Liaoning Cancer Hospital & Institute, No. 44 Xiaoheyan Road, Dadong District, Shenyang, 110001 Liaoning Province China; 3 Department of Clinical Epidemiology and Center of Evidence Based Medicine, The First Hospital of China Medical University, Shenyang, Liaoning Province China; 4 Department of Library, Cancer Hospital of China Medical University/Liaoning Cancer Hospital & Institute, Shenyang, Liaoning Province China; 5 Department of Medical Management, Cancer Hospital of China Medical University/Liaoning Cancer Hospital & Institute, Shenyang, Liaoning Province China

**Keywords:** Childhood central nervous-system tumors, Incidence, Mortality, Clinical-pathological characteristics, Prediction

## Abstract

**Background:**

Central nervous system (CNS) tumors are the most common solid tumors in children and the leading cause of cancer-related death in the latter. Currently, the incidence rate exceeds that of leukemia and ranks first in the incidence of malignant tumors in children.

**Methods:**

The epidemiological data on childhood CNS tumors were collected from the Chinese Cancer Registry Annual Report. The annual percent change (APC) of incidence and mortality-rate changes were estimated via Joinpoint regression. Due to a lack of pertinent data, we performed a system review on the clinical-pathological characteristics in Chinese publications.

**Results:**

There was no significant increase in the incidence rate (APC: -0.1, 95% CI: -1.5 to 1.3), but there was a significant increase in the mortality rate (APC: 1.8, 95% CI: 0.3 to 3.4) for childhood CNS tumors. In the subgroup analysis, there were significant increases in both the incidence and mortality rates in rural areas (APC in the incidence: 6.2, 95% CI: 2.4 to 10.2; APC in mortality: 4.4, 95% CI: 0.4 to 8.4). The most common location and type of childhood CNS were, respectively, the cerebral hemisphere (25.5%, 95% CI: 21.7% to 29.4%) and astrocytomas (26.8%, 95% CI: 23.9% to 29.6%).

**Conclusions:**

The epidemiological trends, and the relevant prediction, highlighted the need to pay continual attention to childhood CNS tumors, and the clinicopathology evinced its own distinctive characteristics. Timely detection and effective treatment must be further promoted regarding childhood CNS tumors with a view to decreasing the disease burden, especially in rural areas.

**Supplementary Information:**

The online version contains supplementary material available at 10.1186/s12885-024-11883-w.

## Background

In comparison with adult cancers, childhood cancers are less common. Nonetheless, the poor prognosis for childhood cancers generates a serious impact on quality of life, especially in the case of childhood central nervous-system (CNS) tumors [1]. Currently, the incidence rate of childhood CNS tumors exceeds that of childhood leukemia, as reflected in the 2020 CBTRUS Statistical Report. Childhood CNS tumors have become the most common cause of cancer death in children, and the leading cause of childhood cancer mortality [2]. Conversely, while many studies have focused on the epidemiological trends and clinicopathological characteristics of adult cancers, few have focused on the status of childhood cancers in China. Although one study did report differences in the incidence rate of CNS between Chinese and American children, the results did not represent the overall situation in China, because the relevant cohort merely addressed Hong Kong [3].

The common locations and types of childhood CNS tumor differ from those in adults. The epidemiology of childhood CNS tumors was found to be affected by the location and pathological type of the lesion [4]. Moreover, the low specificity of CNS tumors in children can easily lead to misdiagnosis or missed diagnosis. Our previous research systematically analyzed the epidemiological changes of CNS tumors in all age groups in China [5]. ​We found a lack of relevant research on the epidemiological and clinicopathological patterns of childhood CNS tumors within the country. A comprehensive analysis of such tumors is urgently required, to provide medical evidence for future prevention and multimodal treatment approaches.

We have, therefore, conducted this study to describe the epidemiological trend from registry data, and for all cancer cases by sex, age, and area. Due to lack of data regarding the clinicopathological characteristics of childhood CNS tumors, we have performed a system review to analyze the locations and types of the tumors.

## Methods

### Data source and subjects

Thus far, the Chinese Cancer Registry Annual Report has released cancer data for the period 2005 to 2017. In our study, the incidence and mortality data for childhood CNS tumors (0–19 age group) were collected from the same Report, which by 2017 had addressed approximately 31.39% of the Chinese population. ​The changes in the number of registries, and the coverage of the population from 2005 to 2017, are detailed in Table S1, and the distribution of cancer registries in China is shown in Figure S1. With the continuous improvement of economic conditions and tumor-monitoring capacity in China, the number of monitoring units is increasing, the quality of monitoring data is improving, and the coverage of the population is also rising. At present, the Chinese Cancer Registry Annual Report comprehensively reflects the development of cancer incidence and death within the country. The resource includes all individuals diagnosed with, or dying from, childhood CNS tumors (ICD-10 codes C70 ~ C72, D32 ~ D33 and D42 ~ D43) between 1 January and 31 December each year.

We extracted the population sizes and incidence/mortality cases for four age groups (0 years, 1–4 years, 5–9 years, 10–14 years, and 15–19 years). Because the incidence/mortality rate in the 0-year age group was too low for our purposes, we combined the related data in the 0-year age group with those for the 1–4-year age group. Incidence rates, mortality, and the annual percent change (APC) in each age group were calculated to capture the trend from 2005 to 2017.

This study had been approved by the Ethics Committee of Liaoning Cancer Hospital. Because the data for our study were derived from the Chinese Cancer Registry Annual Report (2005 to 2017), and the related published article, our study does not involve specific research objects, sensitive human information and data, or commercial interests, and an application for ethical approval was thus waived.

### Search strategy

The Cancer Registry Annual Report did not provide the required information on childhood CNS-tumor positions and subtypes. Consequently, we performed a system review to analyze the locations and types of these tumors. Multiple literature databases (PubMed, Web of Science, China Science and Technology Journal Database (CQVIP), China National Knowledge Infrastructure (CNKI), and Wanfang Data) were searched via an established search strategy. This latter included (1) the key words “central nervous system tumors,” “brain tumor(s),” “brain neoplasm(s),” “spinal cord tumor(s),” “spinal cord tumor(s),” and “spinal cord neoplasm” for searches on PubMed and Web of Science. Meanwhile (2), the above terms were set as subject headings (including title, abstract, and keyword) for searches within the Chinese databases. The searches included all relevant studies published prior to June 30, 2023. All the retrieved references were restricted to “child” and “Chinese population.” We conducted this study, and we reported the related results, based on the guidelines of the Preferred Reporting Items for Systematic Reviews and Meta-Analyses (PRISMA) statement, as issued in 2020 (Table S2). We also registered our review on the Open Science Framework (10.17605/OSF.IO/EX76Z.).

### Study selection

We created an electronic library with Endnote bibliographic software (version X6; Thomson Reuters, Inc., Philadelphia, PA), and all pertinent studies were retrieved from this electronic library. After duplicate studies were identified via the software, two authors (XM W, B Z) independently screened the titles/abstracts and full texts of the remaining studies. When disagreements occurred between the authors, a further author (CY W) was consulted, and agreement was reached by discussion.

The criteria for inclusion were as follows: (1) observational studies on primary tumor; (2) studies evincing a specific research time period and area; (3) a clear diagnostic criterion; (4) all cases being examined via imaging, and evincing a clear location, pathological type and grade; (5) when two or more studies came from the same population, the most recent article, or the article with the largest sample size, was selected. The criteria for exclusion were as follows: (1) studies failing to fulfil the above inclusion criteria; (2) the publication type was that of a review, conference abstract, or case report; (3) the data were not derived from the population, being derived (e.g.) from animal experiments. The International Classification of Childhood Cancer, third edition (ICCC-3), was used as a classification system to distinguish the pathological types of childhood CNS tumor.

### Data extraction and quality assessment

We extracted basic information (First author, Year of publication, Study design, Time interval of case collection, Hospital of diagnosis and treatment, Source of cancer cases, Diagnostic criteria, and Number of subjects) from the included studies. To avoid the inclusion of duplicate cases, we also used three factors (Time interval of case collection, Hospital of diagnosis and treatment, Source of cancer cases) to identify the latter. The quality of the included studies was independently assessed by two authors (XM W, B Z) via a cross-sectional/prevalence study-quality assessment [6]. The detailed contents of the latter are shown in Tables S3 and S4.

### Statistical analyses

The incidence and mortality of childhood CNS tumors were classified by sex, age group (0–4, 5–9, 10–14 and 15–19), and area (urban or rural). Joinpoint software (Version 4.7.0.0) was used to assess the magnitude, and direction of trends over time, for incidence and mortality. The APC was calculated by means of generalized linear models, assuming a Poisson distribution. The Monte Carlo permutation method was deployed to assess the tests of significance, and the overall asymptotic significance level was maintained via a Bonferroni correction. The overall summary estimate for each clinicopathological characteristic was calculated via a random-effects model (DerSimonian-Laird) in the meta-analysis. Heterogeneity was assessed via I-square statistics (I^2^) and Cochran Q-statistics. The meta-analysis was performed using R software (Version 4.1.1). *P* < 0.05 was defined as statistically significant.

## Results

### Trends in the incidence and mortality rates of childhood CNS tumors

**Fig. 1:**
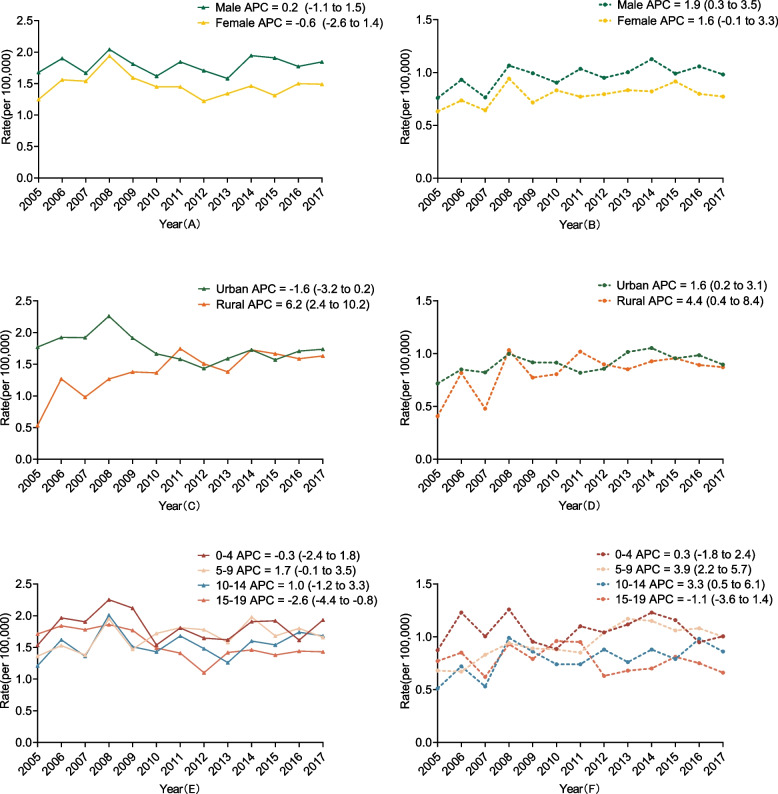
The trends of incidence and mortality rates of Childhood CNS tumors in China from 2005 to 2017. **A** The trend of the incidence rate of Childhood CNS tumors by sex in China from 2005 to 2017. **B** The trend of the mortality rate of Childhood CNS tumors by sex in China from 2005 to 2017. **C** The trend of the incidence rate of Childhood CNS tumors by area in China from 2005 to 2017. **D** The trend of the mortality rate of Childhood CNS tumors by area in China from 2005 to 2017. **E** The trend of the incidence rate of Childhood CNS tumors by age group in China from 2005 to 2017. **F** The trend of the mortality rate of Childhood CNS tumors by age group in China from 2005 to 2017

In China, from 2005 to 2017, a total of 8216 childhood CNS tumors were diagnosed among children aged 0 to 19. There was no significant increase in the incidence rate (APC: -0.1, 95% CI: -1.5 to 1.3). In terms of the area analysis, the incidence rate in rural areas increased significantly (APC: 6.2, 95% CI: 2.4 to 10.2), while no significant change was found in urban areas (APC: -1.6, 95% CI: -3.2 to 0.2). There was a significant decrease in the 15–19-year age group (APC: -2.2, 95% CI: -4.4 to -0.8). The incidence rate in males was higher than that in females, and the incidence in urban areas was also higher than that in rural areas (Fig. 1A, C, E and Table S5).

The number of childhood CNS tumor deaths was 4577. There was a significant increase in the mortality rate (APC:1.8, 95% CI: 0.3 to 3.4). There were also significant increases in CNS mortality from 2005 to 2017, in both the 5–9-year age group and the 10–14-year age group (APC in 5–9-year age group: 3.9, 95% CI: 2.2 to 5.7; APC in 10–14-year age group: 3.3, 95% CI: 0.5 to 6.1). As with the incidence rate, the mortality rate decreased with age. In terms of sex, the mortality rate in males increased significantly (APC: 1.9, 95% CI: 0.3 to 3.5), while no significant change was found in females (APC: 1.6, 95% CI: 0.1 to 3.3), and the mortality rate in males was also higher than that in females. As regards the area analysis, the mortality rate increased significantly in both urban and rural areas (APC in urban:1.6, 95% CI: 0.2 to 3.1; APC in rural: 4.4, 95% CI: 0.4 to 8.4). The mortality rate in urban areas was higher than that in rural areas. The extent of the increase in rural areas was, however, higher than that in urban areas, and the two gradually converged from 2005 to 2017 (Figs. 1B, D and F, and Table S5).

### The clinicopathological characteristics of childhood CNS tumors

**Fig. 2:**
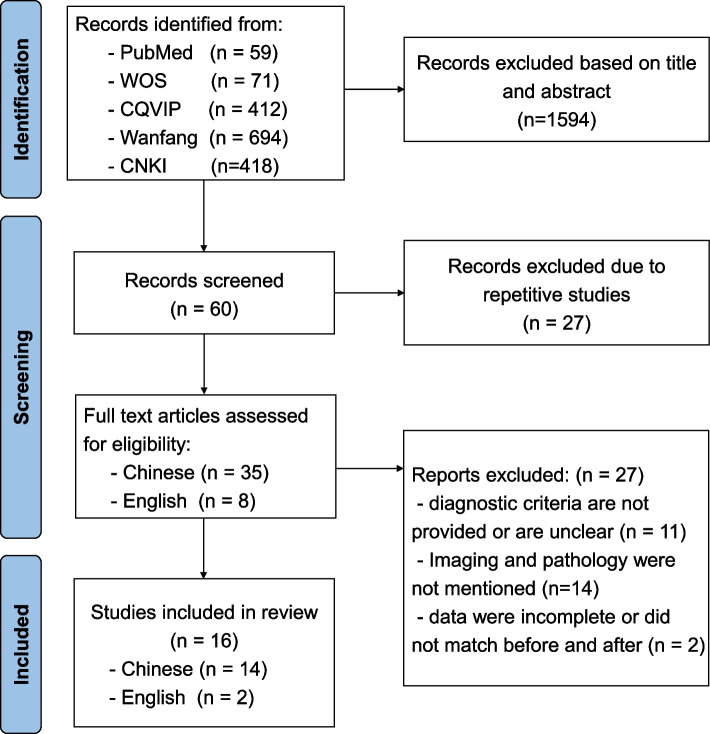
The study selection process in our meta-analysis

In terms of literature collection, 1654 potential studies were searched, across multiple databases. After a review of the titles and abstracts, 1594 studies were excluded. Of the remaining 60 studies, we found that 27 studies had duplicate cases, while studies with larger sample sizes and higher quality were included. Following a careful review of the full texts, 27 studies were excluded. In total, 16 studies satisfied the inclusion criteria for our meta-analysis [7–22]. The study-selection process, and the results from the literature search, are shown in Fig. 2. Table S6 shows the characteristics of the included studies within the meta-analysis. All the included studies evinced a cross-sectional design.

**Fig. 3:**
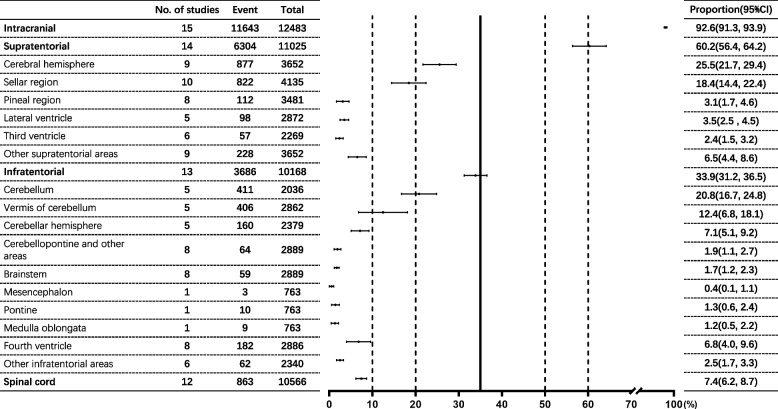
The proportion of Childhood CNS tumor location by meta-analysis

**Fig. 4:**
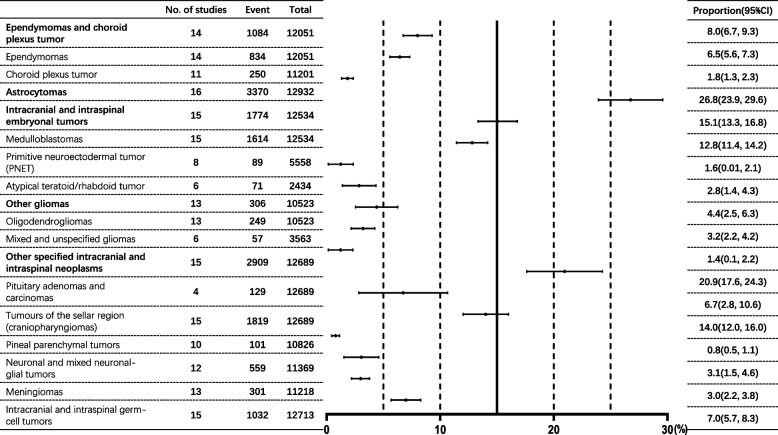
The proportion of Childhood CNS tumor type by meta-analysis

A total of 12,932 children with CNS tumors were addressed by the 16 studies. Among the total population of included children, 56.4% were males (95% CI: 51.7% to 61.0%), and the male-to-female ratio was approximately 1.28:1. Regarding the age-group analysis, the proportion of low-grade tumors increased by age, but the ratio of high-grade and low-grade tumors decreased by age (Table 1). In terms of the analysis of childhood CNS-tumor location, intracranial tumors accounted for 92.6% (95% CI: 91.3% to 93.9%), while spinal-cord tumors accounted for only 7.4% (95% CI: 6.2% to 8.7%), with a ratio of 13.2:1. The ratio of supratentorial tumors to infratentorial tumors was 1.71:1. Among supratentorial tumors, the cerebral hemisphere (25.5%, 95% CI: 21.7% to 29.4%) and sellar region (18.4%, 95% CI: 14.4% to 24.4%) were the main tumor locations, accounting for 67.7% of supratentorial tumors. Among infratentorial tumors, meanwhile, the cerebellum (20.8%, 95% CI: 16.7% to 24.8%) and the vermis of cerebellum (12.4%, 95% CI: 6.8% to 18.1%) were the main tumor locations, accounting for 75.0% of infratentorial tumors (Fig. 3 and Table S7). In the pathological types of childhood CNS tumor, the top five pathological types were astrocytomas (26.8%, 95% CI: 23.9% to 29.6%), tumors of the sellar region (craniopharyngiomas) (14.0%, 95% CI: 12.0% to 16.0%), medulloblastomas (12.8%, 95% CI: 11.4% to 14.2%), intracranial and intraspinal germ-cell tumors (7.0%, 95% CI: 5.7% to 8.3%) and pituitary adenomas and carcinomas (6.7%, 95% CI: 2.8% to 10.6%) (Fig. 4 and Table S8).

**Table 1:** The proportion of low-grade and high-grade childhood CNS tumors in each age group

Age group, years	Low grade (%)	High grade (%)	Ratio (High grade/ Low grade)
0–3	6.9(2.7, 11.1)	7.1(4.4, 9.9)	1.03
4–6	9.2(7.4, 10.9)	9.6(5.6, 13.5)	1.04
7–9	11.4(6.4, 16.5)	11.4(9.6, 13.2)	1.00
10–12	12.2(10.3, 14.0)	9.9(7.6, 12.1)	0.81
13-	13.4(8.0, 18.7)	9.0(5.4, 12.6)	0.67
Total	53.0(45.6, 60.3)	47.1(39.9, 54.3)	0.89

## Discussion

The incidence and mortality trends for childhood CNS tumors in China have rarely been reported. In our study, conversely, we systematically analyzed the relevant data on childhood CNS tumors. There was a significant increase in the mortality rate, but no significant changes in the incidence rate from 2005 to 2017. Similar incidence and mortality trends were observed between males and females. Nonetheless, in China, there were significant increases in both the incidence and mortality rate in rural areas, while the incidence and mortality rates in rural areas were close to those in urban areas by 2017. This change may be caused by the shrinking gap between urban and rural socioeconomic conditions and health-care levels.

With the discovery of the increased mortality rate, we noticed a difference in the proportion of lesion sites and pathological types for childhood CNS tumors. In terms of the clinicopathological characteristics of childhood CNS tumors in China, due to a lack of relevant data for the country, we collected pertinent articles and used the meta-analysis to pool the associated data. Recent studies have found that most childhood CNS tumors are, in fact, low-grade tumors [23]. In China, the proportion of low-grade tumors increased by age, and it was higher than the proportion of high-grade tumors. Conversely, the ratio of high-grade and low-grade childhood CNS tumors decreased by age, and these figures were different from those in the USA [2].

In our study, astrocytoma was the most common type of childhood CNS tumor, followed by craniopharyngioma and medulloblastoma. Reportedly, high birth weight has been one of the risk factors for astrocytoma and embryonic tumors [24]. In the more economically developed areas of China, the incidence of high birth weight has exceeded 10% [25], and this can partly explain the high incidence of CNS among children in urban areas. Gliomas also have a high incidence rate in adults, but meningiomas and pituitary tumors were the most common non-malignant tumors in adults [26]. The incidence of childhood astrocytoma was partially attributed to cancer predisposition syndromes [27]. Pilocytic astrocytoma comprised the most common form of CNS tumor in patients with neurofibromatosis type 1 (NF1), accounting for approximately 49% of cases [28]. It was noteworthy, nevertheless, that NF1-associated pilocytic astrocytoma evinced a better prognosis in children than sporadic pilocytic astrocytoma [29]. Conversely, the clinical monitoring and treatment strategies for NF1-derived CNS tumors are still in the process of optimization [30]. Timely management of NF1 patients, meanwhile, prevented the occurrence of childhood CNS tumors. In addition, parents with higher educational levels were associated with a higher risk of CNS tumors among their children [31]. Large-scale childhood CNS tumor data from China will be needed to explore the issue in the future.

Regarding tumor location, as with the results of childhood CNS tumors in previous studies [32–34], our study found that supratentorial tumors were more common than infratentorial tumors. Meanwhile, the cerebral hemisphere and sellar region were common locations of supratentorial tumors, and the cerebellum and vermis of cerebellum were common locations for infratentorial tumors. Nonetheless, some common subtypes of childhood CNS tumor in China are inconsistent with those in other countries and regions. Generally, it has been considered that the cerebral hemisphere is the most common site of intracranial tumors in adults (this being relatively rare in children), but in recent years, some studies have reported that the proportion of supratentorial tumors is roughly the same as the proportion of infratentorial tumors, even though the proportion of supratentorial tumors is slightly higher [4, 35]. Our results also showed that the cerebral hemisphere was the most common site of intracranial tumors in children, indicating a gradual diversification trend in intracranial tumors among the latter. Due to differences in pathological type and location, childhood CNS tumors in China evinced their own characteristics. In future, we should consider the key locations and types of childhood CNS tumor when we address medical investment for diagnosis and treatment.

The symptoms of CNS tumors in children lack specificity, and they are difficult to identify and diagnose in a timely manner [36]. Supratentorial tumors, which are mainly composed of cerebral-hemisphere tumors and sellar-region tumors, can damage the normal functions of the cerebral-hemisphere, thalamus and sellar region. The main early symptoms of supratentorial tumors are limb weakness, visual-field defects, epileptic seizures, slow reactions, etc. [37]. Most of the infratentorial tumors were found to be cerebellar tumors, and this may be related to the high incidence of neuroepithelial tumors. The main symptoms of infratentorial tumors are vomiting and dizziness [37]. The early symptoms are not serious, and because of the poor expressive abilities of very young children, infratentorial tumors are often ignored or misdiagnosed. This delays treatment.

In the last 20 years, in the USA, there has been a significant decline in the mortality rate and no change in incidence rates, while survival rates have increased by 16% [38]. In fact, the disease burden of childhood CNS tumors has decreased significantly in the USA. This reflects the former, substantial gap in the diagnosis and treatment of childhood CNS tumors between China and more developed countries. Due to the unique characteristics of age, symptoms, and prognosis, childhood CNS tumor patients require specialized professional pediatric care during the treatment and care process [39, 40]. Nonetheless, in low- and middle-income countries, the numerical proportion of childhood CNS tumor patients to professional pediatric neurosurgeons has not yet reached optimal levels. Thus, there has been a lack of experienced health-care professionals [41].

For these reasons, sufficient and professional pediatric neuro-oncology centers, as well as multidisciplinary services staffed by trained surgeons in line with recognized treatment protocols, could not be provided in China or other, less-developed states [42, 43]. The lack of suitable diagnostic conditions often caused delayed diagnoses for children [44], thus affecting the incidence rate [39]. In addition, many childhood CNS tumors required advanced treatment methods. On the one hand, due to high operating costs, commercial medical companies were not willing to conduct advanced clinical trials in low- and middle-income countries [45]. Indeed, with advances in molecular-diagnostic and targeted-therapy technologies, the gap in professional care provision for children became increasingly wide. On the other hand, in order to access advanced treatment technology, patients and their families were often transferred to economically developed, but geographically distant, countries for medical treatment. While such families would benefit from advanced technology, they also faced high treatment costs, a problem exacerbated by insurance policies that did not provide appropriate funding. These issues undoubtedly led to the abandonment of treatment, thus increasing the mortality rate [46]. Therefore, while one may raise awareness of cancer prevention and treatment among Chinese families, the capacities of domestic professional pediatric care, and the question of medical costs, cannot be ignored.

Some limitations of our study should be noted. First, the data collection in cancer registries in China was carried out in accordance with unified steps and procedures. The establishment of cancer registration points has been restricted by regional medical and economic disparities, so coverage of the entire Chinese population could not be guaranteed. The fact that only around 1/3 of Chinese population data was covered in this way caused a certain degree of selection bias in our results. As the most authoritative cancer-registration information source in China, however, the Cancer Registry Annual Report provides the largest available reservoir of population information, so our research results are still representative.

Second, in the Chinese Cancer Registry Annual Report, we could not extract information on treatment type, time to diagnosis and treatment, etc. Thus, we only analyzed the effects of age, gender, and rural/urban disparity. This was a limiting factor for our results. Third, due to the lack of childhood CNS tumor-type information in Chinese cancer reports, we performed a system review to analyze the data of relevant articles, in order to reflect childhood CNS-tumor positions and subtypes. The results from our meta-analysis merely provided an estimate. Still, this reflected the general situation regarding childhood CNS tumor typologies, and the meta-analysis method was a suitable solution for our study. Therefore, after a strict inclusion and exclusion process, we used the cross-sectional/prevalence study quality assessment to assess the quality of the included studies. We found that the scores of all the included studies were above 9, signifying high quality.

Fourth, in the inclusion criteria for our systematic review, we restricted the location and pathological type of lesions to those confirmed by imaging. This may have caused some cases, which had not undergone biopsy or resection, to be overlooked. Fifth, we failed to distinguish between benign and malignant tumors in our systematic review. Future studies, with large sample sizes from multiple centers in China, are thus needed to provide further support.

## Conclusion

There was a significant increase in the incidence and mortality rate of childhood CNS tumors in rural areas, which may be the main reason for the increased burden of childhood CNS tumors. The clinicopathology of childhood CNS tumors in China evinced its own, particular characteristics. The latter must be addressed, in order to improve the treatment of childhood CNS tumors in terms of key positions and types. It is thus essential to formulate preventive and control measures suitable for childhood CNS tumors in China.

### Supplementary Information

**Additional file 1: Table S1.** The information of cancer registries in China between 2005 and 2017. **Table S2.** The PRISMA-2020 Checklist. **Table S3.** The cross-sectional/prevalence Study quality assessment. **Table S4.** The cross-sectional/prevalence Study quality assessment on our included studies. **Table S5.** The results by join point regression on the incidence and mortality rate of Childhood CNS tumors by age, sex, area, and period between 2005 and 2017. **Table S6.** The characteristics of the included studies in the meta-analysis. **Table S7.** The proportion of CNS tumor location by meta-analysis. **Table S8.** The proportion of CNS tumor type by meta-analysis. **Figure S1.** Distribution of cancer registries in China.

## Data Availability

All our research datasets were extracted from the China Cancer Registry Annual Report from 2005 to 2017, which were all publication of books. The annual report we reference can be get in the Global Health Data Exchange (GHDx) online repository, https://ghdx.healthdata.org/series/china-national-central-cancer-registry. Readers can access the data used in our study from the Supplementary materials.

## Abbreviations

CNS: Central nervous system
CQVIP: China Science and Technology Journal Database
CNKI: China National Knowledge Infrastructure
APC: Annual percent change
PRISMA: Preferred Reporting Items for Systematic Reviews and Meta-Analyses
BCG: Bacille Calmette-Guérin
ICCC-3: International Classification of Childhood Cancer, Third edition
NF1: Neurofibromatosis type 1
