# Supplementary material for: A system review of central nervous system tumors on children in China: epidemiology and clinical characteristics
Source: BMC Cancer. 2024 Jan 27;24:138. doi: 10.1186/s12885-024-11883-w (PMC10821253; doi:10.1186/s12885-024-11883-w)
Supplement: Supplementary file 1 — Additional file 1: Table S1. The information of cancer registries in China between 2005 and 2017. Table S2. The PRISMA-2020 Checklist. Table S3. The cross-sectional/prevalence Study quality assessment. Table S4. The cross-sectional/prevalence Study quality assessment on our included studies. Table S5. The results by join point regression on the incidence and mortality rate of Childhood CNS tumors by age, sex, area, and period between 2005 and 2017. Table S6. The characteristics of the included studies in the meta-analysis. Table S7. The proportion of CNS tumor location by meta-analysis. Table S8. The proportion of CNS tumor type by meta-analysis. Figure S1. Distribution of cancer registries in China. [file 12885_2024_11883_MOESM1_ESM.docx]

Supplemental Materials

**A system review of central nervous system tumors on children in China: epidemiology and clinical characteristics**

**Part 1: Supplementary methods**

In order to scientifically and truly reflect the current situation of China's urban and rural population and socioeconomic development, we have formulated standards for urban and rural statistical classification based on the provisions of China's census.

**The criteria for urban are as follows:**

1. If the population density of a city's administrative area is 1,500 people/km2 or more, the urban area is the entire administrative area under the jurisdiction of the district;
2. If the population density of a municipal district is less than 1,500 people per square kilometer, the urban area is the residence of the municipal district people's government and the area of other street offices under the jurisdiction of the district;

3. The townships of the towns where the county and county-above (excluding city) people's governments and administrative offices are located, and the townships of other towns.

**The criteria for rural are as follows:**

1. A market town refers to a non-administrative town located in the seat of the people's government of a township or ethnic township, and which has been developed from a market and serves as a center of economic, cultural, and living services for a certain region of rural areas, as confirmed by the people's government of the county.
2. Rural areas refer to areas outside the towns.

**Part 2 : Supplementary Tables and Figures**

**Table S1** The information of cancer registries in China between 2005 and 2017

| **Year** | **All** | | |  | **Urban** | | |  | **Rural** | | |
| --- | --- | --- | --- | --- | --- | --- | --- | --- | --- | --- | --- |
|  | **No. of registries** | **Total(millions)**  **(%)^a^** | **Population aged 0-19 (millions)(%)^b^** |  | **No. of registries** | **Total(millions)**  **(%)^b^** | **Population aged 0-19 (millions)(%)^c^** |  | **No. of registries** | **Total(millions)**  **(%)^b^** | **Population aged 0-19 (millions)(%)^d^** |
| 2005 | 45 | 54.92 (4.20) | 11.89 (21.65) |  | 20 | 40.67 (74.06) | 7.96 (19.57) |  | 25 | 14.27 (25.94) | 3.93 (27.54) |
| 2006 | 49 | 59.56 (4.53) | 12.39 (20.80) |  | 21 | 46.55 (78.16) | 8.84 (18.99) |  | 28 | 13.00 (21.84) | 3.55 (27.31) |
| 2007 | 48 | 59.80 (4.53) | 12.34 (20.64) |  | 20 | 44.60 (74.59) | 8.17 (18.32) |  | 28 | 15.20 (25.41) | 4.16 (27.37) |
| 2008 | 56 | 66.13 (6.21) | 12.60 (19.05) |  | 26 | 52.15 (78.86) | 9.21 (17.66) |  | 30 | 13.98 (21.14) | 3.38 (24.18) |
| 2009 | 104 | 85.47 (6.40) | 16.95 (20.43) |  | 46 | 57.48 (67.26) | 10.35 (18.01) |  | 58 | 27.98 (32.74) | 6.59 (23.55) |
| 2010 | 219 | 124.65 (9.29) | 25.47 (20.42) |  | 92 | 79.98 (64.17) | 14.90 (18.63) |  | 127 | 44.66 (35.83) | 10.56 (23.65) |
| 2011 | 234 | 145.74 (10.82) | 29.76 (20.15) |  | 98 | 87.52 (60.06) | 16.13 (18.43) |  | 136 | 58.21 (39.94) | 13.26 (22.78) |
| 2012 | 261 | 198.06 (14.63) | 39.91 (15.97) |  | 100 | 100.45 (50.72) | 17.57 (17.49) |  | 161 | 97.61 (49.28) | 22.34 (22.89) |
| 2013 | 347 | 287.28 (21.11) | 45.88 (15.97) |  | 126 | 111.59 (49.27) | 19.50 (17.47) |  | 221 | 114.89 (50.73) | 26.38 (22.96) |
| 2014 | 449 | 288.24 (21.07) | 58.51 (20.30) |  | 160 | 144.06 (49.98) | 25.86 (17.95) |  | 289 | 144.18 (50.02) | 32.65 (22.65) |
| 2015 | 501 | 309.55 (22.52) | 65.16 (21.05) |  | 174 | 148.80 (48.07) | 27.98 (18.80) |  | 327 | 160.74 (51.93) | 19.91 (12.39) |
| 2016 | 682 | 381.56 (27.60) | 79.13 (20.74) |  | 432 | 192.62 (50.48) | 36.47 (18.93) |  | 250 | 188.93 (49.52) | 42.65 (22.57) |
| 2017 | 821 | 436.33 (31.16) | 93.27 (21.38) |  | 523 | 213.24 (48.87) | 42.29 (19.83) |  | 298 | 223.09 (51.13) | 50.97 (22.85) |

^a^ Proportion of the total population at the end of that year.

^b^ Proportion of the total registries at that year.

^c^ Children aged 0-19 years as a proportion of the urban registries at that year.

^d^ Children aged 0-19 years as a proportion of the rural registries at that year.

**Table S2** The PRISMA-2020 Checklist

| **Section and Topic** | **Item #** | **Checklist item** | **Location where item is reported** |
| --- | --- | --- | --- |
| **TITLE** | | |  |
| Title | 1 | Identify the report as a systematic review. | 1 |
| **ABSTRACT** | | |  |
| Abstract | 2 | See the PRISMA 2020 for Abstracts checklist. | 2 |
| **INTRODUCTION** | | |  |
| Rationale | 3 | Describe the rationale for the review in the context of existing knowledge. | 3 |
| Objectives | 4 | Provide an explicit statement of the objective(s) or question(s) the review addresses. | 3 |
| **METHODS** | | |  |
| Eligibility criteria | 5 | Specify the inclusion and exclusion criteria for the review and how studies were grouped for the syntheses. | 4-6 |
| Information sources | 6 | Specify all databases, registers, websites, organisations, reference lists and other sources searched or consulted to identify studies. Specify the date when each source was last searched or consulted. | 4-6 |
| Search strategy | 7 | Present the full search strategies for all databases, registers and websites, including any filters and limits used. | 4-6 |
| Selection process | 8 | Specify the methods used to decide whether a study met the inclusion criteria of the review, including how many reviewers screened each record and each report retrieved, whether they worked independently, and if applicable, details of automation tools used in the process. | 4-6 |
| Data collection process | 9 | Specify the methods used to collect data from reports, including how many reviewers collected data from each report, whether they worked independently, any processes for obtaining or confirming data from study investigators, and if applicable, details of automation tools used in the process. | 4-6 |
| Data items | 10a | List and define all outcomes for which data were sought. Specify whether all results that were compatible with each outcome domain in each study were sought (e. g. for all measures, time points, analyses), and if not, the methods used to decide which results to collect. | 4-6 |
|  | 10b | List and define all other variables for which data were sought (e.g. participant and intervention characteristics, funding sources). Describe any assumptions made about any missing or unclear information. | 4-6 |
| Study risk of bias assessment | 11 | Specify the methods used to assess risk of bias in the included studies, including details of the tool(s) used, how many reviewers assessed each study and whether they worked independently, and if applicable, details of automation tools used in the process. | - |
| Effect measures | 12 | Specify for each outcome the effect measure(s) (e.g. risk ratio, mean difference) used in the synthesis or presentation of results. | 4-6 |
| Synthesis methods | 13a | Describe the processes used to decide which studies were eligible for each synthesis (e.g. tabulating the study intervention characteristics and comparing against the planned groups for each synthesis (item #5)). | - |
|  | 13b | Describe any methods required to prepare the data for presentation or synthesis, such as handling of missing summary statistics, or data conversions. | 4-6 |
|  | 13c | Describe any methods used to tabulate or visually display results of individual studies and syntheses. | - |
|  | 13d | Describe any methods used to synthesize results and provide a rationale for the choice(s). If meta-analysis was performed, describe the model(s), method(s) to identify the presence and extent of statistical heterogeneity, and software package(s) used. | 4-6 |
|  | 13e | Describe any methods used to explore possible causes of heterogeneity among study results (e. g. subgroup analysis, meta-regression). | 4-6 |
|  | 13f | Describe any sensitivity analyses conducted to assess robustness of the synthesized results. | - |
| Reporting bias assessment | 14 | Describe any methods used to assess risk of bias due to missing results in a synthesis (arising from reporting biases). | - |
| Certainty assessment | 15 | Describe any methods used to assess certainty (or confidence) in the body of evidence for an outcome. | - |
| **RESULTS** | | |  |
| Study selection | 16a | Describe the results of the search and selection process, from the number of records identified in the search to the number of studies included in the review, ideally using a flow diagram. | 6-8, Fig 2 |
|  | 16b | Cite studies that might appear to meet the inclusion criteria, but which were excluded, and explain why they were excluded. | Fig 2 |
| Study characteristics | 17 | Cite each included study and present its characteristics. | Table S6 |
| Risk of bias in studies | 18 | Present assessments of risk of bias for each included study. | - |
| Results of individual studies | 19 | For all outcomes, present, for each study: (a) summary statistics for each group (where appropriate) and (b) an effect estimate and its precision (e.g. confidence/credible interval), ideally using structured tables or plots. | Table S7, Table S8 |
| Results of syntheses | 20a | For each synthesis, briefly summarise the characteristics and risk of bias among contributing studies. | 6-8 |
|  | 20b | Present results of all statistical syntheses conducted. If meta-analysis was done, present for each the summary estimate and its precision (e. g. confidence/credible interval) and measures of statistical heterogeneity. If comparing groups, describe the direction of the effect. | 6-8 |
|  | 20c | Present results of all investigations of possible causes of heterogeneity among study results. | 6-8 |
|  | 20d | Present results of all sensitivity analyses conducted to assess the robustness of the synthesized results. | - |
| Reporting biases | 21 | Present assessments of risk of bias due to missing results (arising from reporting biases) for each synthesis assessed. | - |
| Certainty of evidence | 22 | Present assessments of certainty (or confidence) in the body of evidence for each outcome assessed. | - |
| **DISCUSSION** | | |  |
| Discussion | 23a | Provide a general interpretation of the results in the context of other evidence. | 7-9 |
|  | 23b | Discuss any limitations of the evidence included in the review. | 7-9 |
|  | 23c | Discuss any limitations of the review processes used. | 7-9 |
|  | 23d | Discuss implications of the results for practice, policy, and future research. | 7-9 |
| **OTHER INFORMATION** | | |  |
| Registration and protocol | 24a | Provide registration information for the review, including register name and registration number, or state that the review was not registered. | - |
|  | 24b | Indicate where the review protocol can be accessed, or state that a protocol was not prepared. | - |
|  | 24c | Describe and explain any amendments to information provided at registration or in the protocol. | - |
| Support | 25 | Describe sources of financial or non-financial support for the review, and the role of the funders or sponsors in the review. | 10 |
| Competing interests | 26 | Declare any competing interests of review authors. | 10 |
| Availability of data, code and other materials | 27 | Report which of the following are publicly available and where they can be found: template data collection forms; data extracted from included studies; data used for all analyses; analytic code; any other materials used in the review. | - |

*From:*  Page MJ, McKenzie JE, Bossuyt PM, Boutron I, Hoffmann TC, Mulrow CD, et al. The PRISMA 2020 statement: an updated guideline for reporting systematic reviews. BMJ 2021;372:n71. doi: 10.1136/bmj.n71

For more information, visit: <http://www.prisma-statement.org/>

**Table S3** The cross-sectional/prevalence study quality assessment

| **Item** | **Yes** | **No** | **Unclear** |
| --- | --- | --- | --- |
| 1) Define the source of information (survey, record review) |  |  |  |
| 2) List inclusion and exclusion criteria for exposed and unexposed subjects (cases and controls) or refer to previous publications |  |  |  |
| 3) Indicate time period used for identifying patients |  |  |  |
| 4) Indicate whether or not subjects were consecutive if not population-based |  |  |  |
| 5) Indicate if evaluators of subjective components of study were masked to other aspects of the status of the participants |  |  |  |
| 6) Describe any assessments undertaken for quality assurance purposes (e.g., test/retest of primary outcome measurements) |  |  |  |
| 7) Explain any patient exclusions from analysis |  |  |  |
| 8) Describe how confounding was assessed and/or controlled. |  |  |  |
| 9) If applicable, explain how missing data were handled in the analysis |  |  |  |
| 10) Summarize patient response rates and completeness of data collection |  |  |  |
| 11) Clarify what follow-up, if any, was expected and the percentage of patients for which incomplete data or follow-up was obtained |  |  |  |

* The methodological quality of the studies included was assessed using an 11-item checklist which was recommended by Agency for Healthcare Research and Quality (AHRQ). An item would be scored ‘0’ if it was answered ‘NO’ or ‘UNCLEAR’; if it was answered ‘YES’, then the item scored ‘1’. Article quality was assessed as follows: low quality = 0–3; moderate quality = 4–7; high quality = 8–11.

**Table S4** The cross-sectional/prevalence Study quality assessment on our included studies

| **First Author** | **First item** | **Second item** | **Third item** | **Fourth item** | **Fifth item** | **Sixth item** | **Seventh item** | **Eighth item** | **Ninth item** | **Tenth item** | **Eleventh item** | **Score** |
| --- | --- | --- | --- | --- | --- | --- | --- | --- | --- | --- | --- | --- |
| Wu, XC[1] | yes | yes | yes | yes | unclear | yes | yes | yes | Yes | yes | no | 9 |
| Xu, XK[2] | yes | yes | yes | yes | unclear | yes | yes | yes | Yes | yes | no | 9 |
| An, X[3] | yes | yes | yes | yes | unclear | yes | yes | yes | yes | yes | no | 9 |
| Li, JZ[4] | yes | yes | yes | yes | yes | yes | yes | yes | yes | yes | no | 9 |
| Ye, YQ[5] | yes | yes | yes | yes | unclear | yes | yes | yes | yes | yes | yes | 10 |
| Tang, XF[6] | yes | yes | yes | yes | unclear | yes | yes | yes | yes | Yes | no | 9 |
| Cui, MM[7] | yes | yes | yes | yes | yes | yes | yes | yes | yes | yes | no | 10 |
| Wang, J[8] | yes | yes | yes | yes | unclear | yes | yes | yes | yes | yes | no | 9 |
| Liu, WD[9] | yes | yes | yes | yes | unclear | yes | yes | yes | yes | yes | yes | 10 |
| Zhu, T[10] | yes | yes | yes | yes | yes | yes | yes | yes | yes | yes | yes | 11 |
| Cui, SM[11] | yes | yes | yes | yes | unclear | yes | yes | yes | yes | yes | no | 9 |
| Shen, WQ[12] | yes | yes | yes | yes | yes | yes | yes | yes | yes | yes | no | 10 |
| Gui, T[13] | yes | yes | yes | yes | unclear | yes | yes | yes | yes | yes | no | 9 |
| Zhou, D[14] | yes | yes | yes | yes | yes | yes | yes | yes | yes | yes | yes | 11 |
| Chen, LG[15] | yes | yes | yes | yes | yes | yes | yes | yes | yes | yes | yes | 11 |
| Chen, H[16] | yes | yes | yes | yes | unclear | yes | yes | yes | yes | yes | unclear | 9 |

**Table S5** The results on the cases, incidence and mortality rate of Childhood CNS tumors by age, sex and area between 2005 and 2017, and its temporal trends from 2005 to 2017

| **Measure** | **Characteristics** | **N (%)** | |  | **Rate (1/10^5^)** | | **2005-2017**  **APC (95 CI %)** |
| --- | --- | --- | --- | --- | --- | --- | --- |
|  |  | **2005** | **2017** |  | **2005** | **2017** |  |
| **Incidence** | | | | | | | |
|  | **Total** | 175 | 1565 |  | 1.47 | 1.68 | -0.1(-1.5, 1.4) |
|  | **Age group, years** | | | | | | |
|  | 0-4 | 28 | 471 |  | 1.53 | 1.93 | -0.3(-2.4, 1.8) |
|  | 5-9 | 34 | 399 |  | 1.36 | 1.66 | 1.7(-0.1, 3.5) |
|  | 10-14 | 40 | 363 |  | 1.21 | 1.68 | 1(-1.2, 3.3) |
|  | 15-19 | 73 | 333 |  | 1.71 | 1.43 | **-2.6(-4.4, -0.8)** |
|  | **Sex** | | | | | | |
|  | Male | 104 | 913 |  | 1.67 | 1.84 | 0.2(-1.1, 1.5) |
|  | Female | 71 | 652 |  | 1.25 | 1.49 | -0.6(-2.6, 1.4) |
|  | **Area** | | | | | | |
|  | Urban | 141 | 735 |  | 1.77 | 1.74 | -1.6(-3.2, 0.2) |
|  | Rural | 34 | 830 |  | 0.53 | 1.63 | **6.2(2.4, 10.2)** |
| **Mortality** | | | | | | | |
|  | **Total** | 83 | 825 |  | 0.69 | 1.01 | **1.8(0.3, 3.4)** |
|  | **Age group, years** | | | | | | |
|  | 0-4 | 16 | 245 |  | 0.84 | 1.00 | 0.3(-1.8, 2.4) |
|  | 5-9 | 17 | 240 |  | 0.68 | 0.86 | **3.9(2.2, 5.7)** |
|  | 10-14 | 17 | 186 |  | 0.51 | 0.66 | **3.3(0.5, 6.1)** |
|  | 15-19 | 33 | 154 |  | 0.77 | 0.66 | -1.1(-3.6, 1.4) |
|  | **Sex** | | | | | | |
|  | Male | 47 | 486 |  | 0.76 | 0.98 | **1.9(0.3, 3.5)** |
|  | Female | 36 | 339 |  | 0.63 | 0.77 | 1.6(-0.1, 3.3) |
|  | **Area** | | | | | | |
|  | Urban | 57 | 380 |  | 0.72 | 0.90 | **1.6(0.2, 3.1)** |
|  | Rural | 26 | 445 |  | 0.41 | 0.87 | **4.4(0.4, 8.4)** |

**Table S6** The characteristics of the included studies in the meta-analysis

| **First Author** | **Year of publication** | **The design of study** | **Time interval of case collection** | **Hospital of diagnosis and treatment** | **Source of cancer cases** | **The diagnostic criteria** | **Number of subjects** |
| --- | --- | --- | --- | --- | --- | --- | --- |
| Wu, XC[1] | 2023 | cross-sectional | 2013.1-2021.12 | The First affiliated Hospital of Xinjiang Medical University | Northwest (Xinjiang) | WHO (2021) classification and diagnostic criteria of central nervous system tumors | 243 |
| Xu, XK[2] | 2022 | cross-sectional | 2015.1-2021.6 | Guangzhou Women and Children's Medical Cancer | South China (Guangdong) | WHO (2016) classification and diagnostic criteria of central nervous system tumors | 483 |
| An, X[3] | 2022 | cross-sectional | 2015.1-2019.12 | 44 hospitals in Beijing | North China  (Hebei, Henan, Beijing) East China (Shandong, Anhui) | WHO (2016) classification and diagnostic criteria of central nervous system tumors | 4951 |
| Li, JZ[4] | 2019 | cross-sectional | 2008.1-2018.12 | The First affiliated Hospital of Xinjiang Medical University | Northwest (Xinjiang) | WHO (2016) classification and diagnostic criteria of central nervous system tumors | 398 |
| Ye, YQ[5] | 2018 | cross-sectional | 2008.1-2017.12 | Xijing Hospital of Air Force Military Medical University | Northwest (Shanxi, Gansu, Qinghai, Ningxia, Xinjiang) | WHO (2016) classification and diagnostic criteria of central nervous system tumors | 839 |
| Tang, XF[6] | 2015 | cross-sectional | 2004.1-2014.8 | Third Military Medical University | Southwest  (Chongqing) | WHO (2007) classification and diagnostic criteria of central nervous system tumors | 221 |
| Cui, MM[7] | 2015 | cross-sectional | 2002.1-2012.12 | The Third Affiliated Hospital of Harbin Medical University | Northeast  (Heilongjiang) | WHO (2007) classification and diagnostic criteria of central nervous system tumors | 163 |
| Wang, J[8] | 2015 | cross-sectional | 2000.10-2014.7 | Sun Yat-sen University cancer center | South China (Guangdong) | WHO (2007) classification and diagnostic criteria of central nervous system tumors | 219 |
| Liu, WD[9] | 2014 | cross-sectional | 2000.1-2012.12 | Provincial Hospital Affiliated to Shandong University and Liaocheng people's Hospital | East China  (Shandong) | WHO (2007) classification and diagnostic criteria of central nervous system tumors | 383 |
| Zhu, T[10] | 2012 | cross-sectional | 1999.1-2009.1 | Tianjin Medical University General Hospital | North China  (Tianjin) | WHO (2007) classification and diagnostic criteria of central nervous system tumors | 468 |
| Cui, SM[11] | 2009 | cross-sectional | 1988.7-2008.6 | Tianjin Huanhu Hospital | North China  (Tianjin) | WHO (2007) classification and diagnostic criteria of central nervous system tumors | 5540 |
| Shen, WQ[12] | 2008 | cross-sectional | 1995.1-2004.12 | Huashan Hospital of Fudan University | East China  (Shanghai) | WHO (2000) classification and diagnostic criteria of central nervous system tumors | 763 |
| Gui, T[13] | 2008 | cross-sectional | 1995.1-2017.12 | Qilu Hospital of Shandong university | East China  (Shandong) | WHO (2007) classification and diagnostic criteria of central nervous system tumors | 330 |
| Zhou, D[14] | 2008 | cross-sectional | 2001.1-2005.12 | Beijing Tiantan Hospital | North China  (Beijing) | WHO (2000) classification and diagnostic criteria of central nervous system tumors | 1485 |
| Chen, LG[15] | 2007 | cross-sectional | 1996.1-2006.12 | Affiliated Hospital of Luzhou Medical College | Southwest (Southern Sichuan) | WHO (2000) classification and diagnostic criteria of central nervous system tumors | 857 |
| Chen, H[16] | 2001 | cross-sectional | 1986.1-1998.12 | Huashan Hospital of Fudan University | East China  (Shanghai) | WHO (1993) classification and diagnostic criteria of central nervous system tumors | 766 |

**Table S7** The proportion of CNS tumor location by meta-analysis

| Location | No. of stuides | Event | Total | Proportion(95%CI) | I^2^（%） | Heterogeneity | p |
| --- | --- | --- | --- | --- | --- | --- | --- |
| Intracranial | 15 | 11643 | 12483 | 92.6 (91.3, 93.9) | 80.2 | 55.54 | <0.001 |
| Supratentorial | 14 | 6304 | 11025 | 60.2 (56.4, 64.2) | 93.2 | 190.84 | <0.001 |
| Cerebral hemisphere | 9 | 877 | 3652 | 25.5 (21.7, 29.4) | 86.4 | 58.93 | <0.001 |
| Sellar region | 10 | 822 | 4135 | 18.4 (14.4, 22.4) | 91.2 | 102.11 | <0.001 |
| Pineal region | 8 | 112 | 3481 | 3.1 (1.7, 4.6) | 88.3 | 59.79 | <0.001 |
| Lateral ventricle | 5 | 98 | 2872 | 3.5 (2.5 , 4.5) | 53.1 | 10.67 | <0.001 |
| Third ventricle | 6 | 57 | 2269 | 2.4 (1.5, 3.2) | 46.5 | 9.34 | <0.001 |
| Other supratentorial areas | 9 | 228 | 3652 | 6.5 (4.4, 8.6) | 86.9 | 61.27 | <0.001 |
| Infratentorial | 13 | 3686 | 10168 | 33.9 (31.2, 36.5) | 83.6 | 73.09 | <0.001 |
| Cerebellum | 5 | 411 | 2036 | 20.8 (16.7, 24.8) | 79.8 | 19.84 | <0.001 |
| Vermis of cerebellum | 5 | 406 | 2862 | 12.4 (6.8, 18.1) | 95.8 | 119.23 | <0.001 |
| Cerebellar hemisphere | 5 | 160 | 2379 | 7.1 (5.1, 9.2) | 71.7 | 14.13 | <0.001 |
| Cerebellopontine and other areas | 8 | 64 | 2889 | 1.9 (1.1, 2.7) | 61.0 | 17.97 | <0.001 |
| Brainstem | 8 | 59 | 2889 | 1.7 (1.2, 2.3) | 27.8 | 9.69 | <0.001 |
| mesencephalon | 1 | 3 | 763 | 0.4 (0.1, 1.1) | -^a^ | -^a^ | -^a^ |
| Pontine | 1 | 10 | 763 | 1.3 (0.6, 2.4) | -^a^ | -^a^ | -^a^ |
| Medulla oblongata | 1 | 9 | 763 | 1.2 (0.5, 2.2) | -^a^ | -^a^ | -^a^ |
| Fourth ventricle | 8 | 182 | 2886 | 6.8 (4.0, 9.6) | 92.4 | 92.64 | <0.001 |
| Other infratentorial areas | 6 | 62 | 2340 | 2.5 (1.7, 3.3) | 26.9 | 6.84 | <0.001 |
| Spinal cord | 12 | 863 | 10566 | 7.4 (6.2, 8.7) | 79.6 | 54.00 | <0.001 |

^a^ The amount of data is small and the statistical error may be too large. This item will not be analyzed.

**Table S8** The proportion of CNS tumor type by meta-analysis

| Type | No. of stuides | Event | Total | Proportion(95%CI) | I^2^（%） | Heterogeneity | p |
| --- | --- | --- | --- | --- | --- | --- | --- |
| Ependymomas and choroid plexus tumor | 14 | 1084 | 12051 | 8.0 (6.7, 9.3) | 81.5 | 70.25 | <0.001 |
| Ependymomas | 14 | 834 | 12051 | 6.5 (5.6, 7.3) | 65.4 | 37.60 | <0.001 |
| Choroid plexus tumor | 11 | 250 | 11201 | 1.8 (1.3, 2.3) | 68.1 | 31.31 | <0.001 |
| Astrocytomas | 16 | 3370 | 12932 | 26.8 (23.9, 29.6) | 91.4 | 174.80 | <0.001 |
| Intracranial and intraspinal embryonal tumors | 15 | 1774 | 12534 | 15.1 (13.3, 16.8) | 84.0 | 87.76 | <0.001 |
| Medulloblastomas | 15 | 1614 | 12534 | 12.8 (11.4, 14.2) | 76.8 | 60.33 | <0.001 |
| Primitive neuroectodermal tumor (PNET) | 8 | 89 | 5558 | 1.6 (0.01, 2.1) | 65.9 | 20.53 | <0.001 |
| Atypical teratoid/rhabdoid tumor | 6 | 71 | 2434 | 2.9(1.3, 4.3) | 81.8 | 27.45 | <0.001 |
| Other gliomas | 13 | 306 | 10523 | 4.4 (2.5, 6.3) | 97.2 | 467.43 | <0.001 |
| Oligodendrogliomas | 13 | 249 | 10523 | 3.2 (2.6, 4.2) | 88.7 | 106.23 | <0.001 |
| Mixed and unspecified gliomas | 6 | 57 | 3563 | 1.4 (0.1, 2.2) | 80.9 | 26.13 | <0.001 |
| Other specified intracranial and intraspinal neoplasms | 15 | 2909 | 12689 | 20.9 (17.6, 24.3) | 95.0 | 297.91 | <0.001 |
| Pituitary adenomas and carcinomas | 4 | 129 | 12689 | 6.7 (2.8, 10.6) | 93.6 | 46.60 | 0.005 |
| Tumours of the sellar region (craniopharyngiomas) | 15 | 1819 | 12689 | 14.0 (12.0, 16.0) | 89.2 | 129.11 | <0.001 |
| Pineal parenchymal tumors | 10 | 101 | 10826 | 0.8 (0.5, 1.1) | 71.9 | 32.02 | <0.001 |
| Neuronal and mixed neuronal-glial tumors | 12 | 559 | 11369 | 3.1 (1.5, 4.6) | 95.7 | 258.69 | <0.001 |
| Meningiomas | 13 | 301 | 11218 | 3.0 (2.2, 3.8) | 81.7 | 65.55 | <0.001 |
| Intracranial and intraspinal germ-cell tumors | 15 | 1032 | 12713 | 7.0 (5.7, 8.3) | 85.9 | 99.44 | <0.001 |


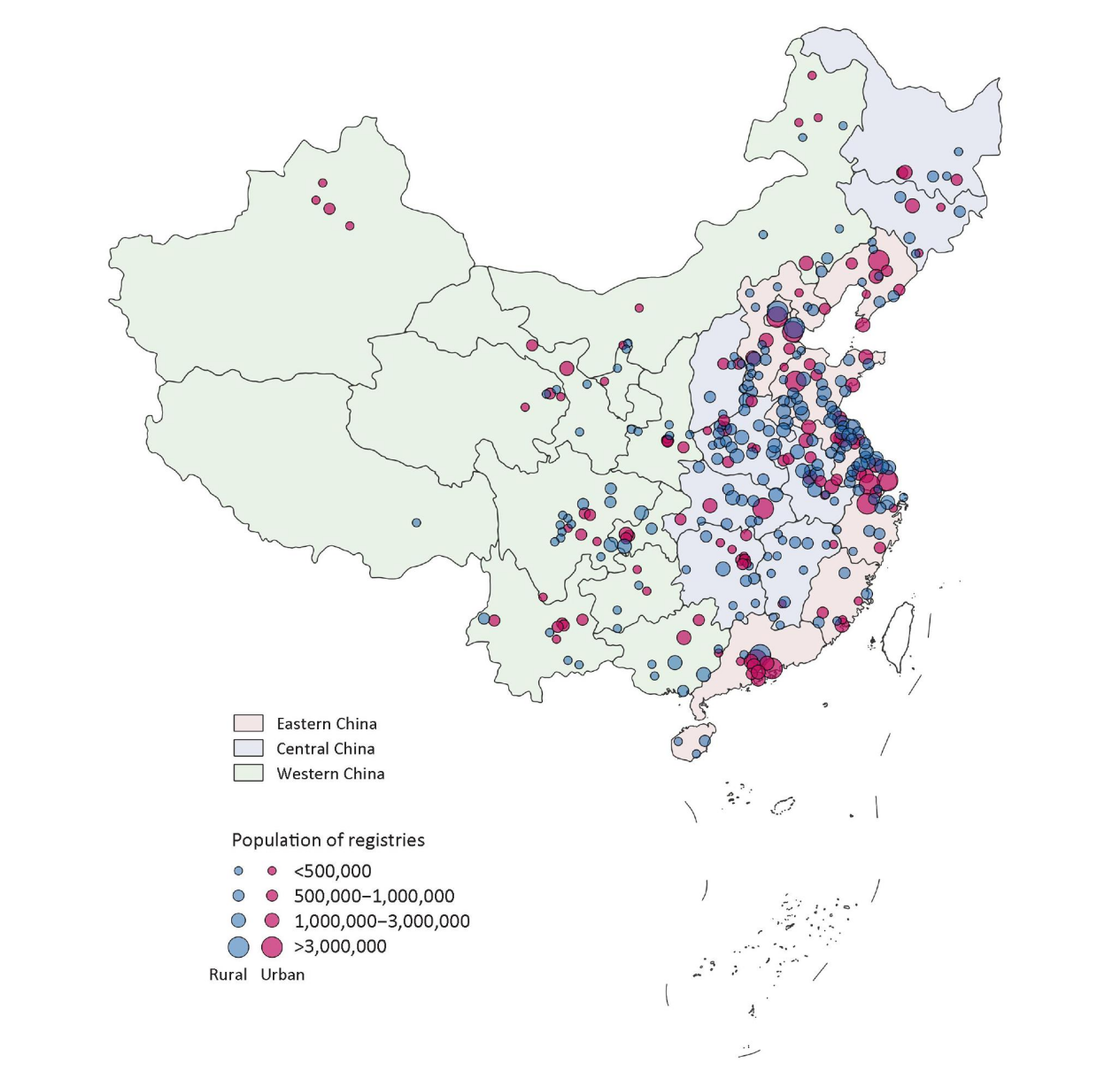


**Figure S1** Distribution of cancer registries in China[17].

**Reference**

1. Wu X, Dangmurenjiafu·Geng, Fan G, Zeng J, Zhao X, Sheng C, et al. Epidemiology of pediatric central nervous system tumors in Uyghur: experience from a single center. Childs Nerv Syst. 2023;39:909–14.

2. Xu X, Li J, Lin J, Chen C, Yang R, Li F. Clinicopathological characteristics of central nervous system tumors in 483 children. Academic Journal of Guangzhou Medical College. 2022;50:51–4.

3. An X, Tian Y, Zheng J, Liu Z, Liu G, Wang J, et al. A disease type review of pediatric central nervous system tumors diagnosed and treated in 44 hospitals in Beijing. Chinese Journal of Neurosurgery. 2022;38:934–40.

4. Li J, Chu H, Miao N, JIao J, Zhang W. Pediatric tumors of nervous system tumors: a clinicopathologic study of 398 cases. J Diag Pathol. 2019;26:333–7.

5. Ye Y, He X, Yang Y, Su X, Kong C, Bai W, et al. Clinical characteristics and epidemiology of intracranial tumor in childhood: a single center survey in northwest of China. Chin J Neurosurg Dis Res. 2018;17:198–202.

6. Tang X, Kong Y, Guo Q. Intracranial tumor in children:a clinicopathological analysis of 221 cases. J Clin Exp Pathol. 2015;31:298–301.

7. Cui M, Dong X. Clinical analysis of central nervous system tumors in children: a report of 163 cases. Journal of Harbin Medical University. 2015;49:533–6.

8. Wang J, Zhao Y, Yang Q, Sai K, Ke C, et al. Clinical analysis of pediatric central nervous system tumors: a retrospective study of 219 cases. Guangdong Medical Journal. 2015;36:2621–4.

9. Liu W. A retrospective and prognostic analysis of pediatrics central nervous system tumors: evaluation of EGFR family gene amplification and overexpression in embryonal tumors and glioblastomas. Doctor. Shandong University; 2014.

10. Tao ZHU, Yun-hu YU, Da-jian Z, Jian-ning Z. Clinical analysis of central nervous system tumors in children, a report of 468 cases. Chinese Journal of Neurosurgery. 2012;28:8–12.

11. Cui S, Qin J, Liu M, Jin S, Han T, Yan S, et al. A statistisc analysis of 5540 cases of central nervous system tumors with WHO classification. Chin J Contemp Neurol Neurosurg. 2009;9:65–9.

12. Shen W. Clinical Analysis of Primary Pediatric Central Nervous System Tumors. Master. Fudan University; 2008.

13. Gui T, Zhang Q, Zhang T, Zhou Z, Liu W, Li H, et al. Central nervous system tumors in childhood: A analysis of 330 cases. J Clin Exp Pathol. 2008;:403–6.

14. Zhou D, Zhang Y, Liu H, Luo S, Luo L, Dai K. Epidemiology of Nervous System Tumors in Children: A Survey of 1,485 Cases in Beijing Tiantan Hospital from 2001 to 2005. Pediatr Neurosurg. 2008;44:97–103.

15. Chen L, Ming Y, Liu L, Li D, Xia X, Gu Y, et al. Epidemiologic survey of intracranial tumors in childhood in southern area of sichuan. Journal of Luzhou Medical College. 2007;:476–9.

16. Chen H, Wu J, Chen J, Zhou L, Zhang F. Central Nervous System Tumors in Children-analysis of 766 cases. Chinese Clinical Neuroscience. 2001;:262–5.

17. Zheng R, Qu C, Zhang S, Zeng H, Sun K, Gu X, et al. Liver cancer incidence and mortality in China: Temporal trends and projections to 2030. Chinese Journal of Cancer Research. 2018;30:571–9.
